# Racial and Ethnic Disparities Associated With the Use of Opioids for Chronic Non-cancer Pain

**DOI:** 10.7759/cureus.102905

**Published:** 2026-02-03

**Authors:** Patrick I Oliorah, Sujit S Sansgiry

**Affiliations:** 1 College of Pharmacy, University of Houston, Houston, USA; 2 Pharmaceutical Health Outcomes and Policy, University of Houston, Houston, USA

**Keywords:** addiction, chronic non-cancer pain, opioids, pain management, race

## Abstract

Chronic non-cancer pain (CNCP) affects a substantial proportion of adults in the United States and is commonly managed using both opioid and non-opioid therapies. This study examined racial and ethnic disparities associated with opioid use for CNCP using nationally representative data. With national efforts and evolving clinical guidelines to reduce opioid use for non-cancer pain, it is not clear if these efforts have helped everyone equally. The objective of the study was to examine racial and ethnic disparities associated with opioid use as a treatment strategy for CNCP. A serial cross-sectional study was conducted using the nationally representative Medical Expenditure Panel Survey (MEPS) data (2016-2021) to estimate opioid and non-opioid treatment choices for CNCP. Racial/ethnic disparities were evaluated by comparing non-Hispanic White patients (whites) with non-Hispanic Black patients (blacks), non-Hispanic Asian/Native American/other/multiple patients (Asians/others), and Hispanic patients. In a sample of 7521 individuals (weighted sample = 14.12M) from 2016 to 2021 with CNCP, our study unveiled that 5.51% utilized opioid therapies while 94.49% utilized non-opioid treatments. Results indicated racial disparities were evident even with declining opioid utilization, as Black individuals exhibited lower estimated odds of opioid utilization (OR = 0.759, 95% CI: 1.240-1.686, p < 0.05) compared to the White population. Other comparisons were not significant. Results revealed significant racial disparities in opioid utilization for CNCP, with Black patients less likely to receive opioid therapy compared to White patients, despite overall declining opioid use.

## Introduction

Pain imposes a greater economic burden than heart disease, cancer, or diabetes in the United States, with annual direct healthcare costs estimated at $261-$300 billion and an additional $299-$355 billion attributed to productivity losses [1]. Recent studies have revealed 22.1% (70M) of individuals in the United States experiencing chronic pain used prescription opioids [2]. Over the past decade, government agencies and healthcare stakeholders have implemented clinical guidelines and policy initiatives aimed at reducing opioid prescribing, which has contributed to reductions in opioid use for pain management [3]. However, it remains unclear if these reductions are consistent across all racial and ethnic groups.

Chronic pain affects multiple dimensions of daily life, including physical functioning, mental well-being, and quality of life, and contributes substantially to long-term morbidity [4]. Nearly 1 in 5 individuals in the United States suffer from chronic non-cancer pain (CNCP) for conditions such as arthritis, headache, migraine, neuropathy, and neck-back pain [5,6]. By focusing on CNCP, the emphasis is on a subset of patients who often experience long-term suffering with complex pain management needs [7]. Unlike cancer pain, which may be treated more aggressively due to its association with terminal illness, CNCP usually presents ethical and medical challenges around long-term opioid use, lifestyle adjustments, and mental health impact [8]. The Centers for Disease Control and Prevention (CDC) analyzed the National Health Interview Survey (NHIS) data to estimate the prevalence of chronic pain in the United States and estimated 20.4% (50.0 million) of U.S. adults had chronic pain [9]. Inadequately treated chronic pain is associated with reduced quality of life, disability, interference with activities of daily living and work, and psychological and cognitive effects, often leading to increased healthcare utilization and productivity losses [10]. The need to treat patients appropriately for chronic pain is a constant struggle for clinicians.

Opioids can be essential for pain management; however, their use is associated with substantial risks, including addiction, cognitive, gastrointestinal, and endocrine effects, and the development of tolerance and dependence [11]. Increased use of opioid pain relievers has contributed to the overall increase in rates of overdose deaths [12]. The CDC has documented substantial increases in overdose deaths involving prescription opioid pain relievers in the United States over time [9]. Pain management for CNCP using non-opioid strategies includes over-the-counter medications such as acetaminophen and nonsteroidal anti-inflammatory drugs (NSAIDs), antidepressants, anticonvulsants, muscle relaxants, physical therapy and exercise programs, and complementary therapies such as acupuncture and massage, with CDC guidelines also highlighting referral to pain specialists and consideration of interventional therapies when appropriate. [3]. The CDC issued the "CDC Guideline for Prescribing Opioids for Chronic Pain" in 2016 [3], which was updated in 2022 [13]. These guidelines recommend non-opioid therapies as first-line treatment for chronic pain and suggest limiting opioid dosages and durations when prescribed [13]. The CDC's 2016 and 2022 guidelines emphasize reducing opioid use, yet no clear data indicate whether this reduction has been consistent across all racial and ethnic demographics [3,13]. Recent research has not explored whether there has been a rise or fall in racial disparities amidst the declining use of opioids, if any. This study aims to evaluate racial/ethnic disparities associated with the use of opioids for CNCP.

## Materials and methods

Study design: data sources

In this retrospective study, the primary data source is the Medical Expenditure Panel Survey (MEPS). MEPS is a panel-based nationally representative survey of the US civilian non-institutionalized population since 1996 [14]. Each MEPS panel collects information on demographic characteristics, prescription medicines, treatments, medical conditions, healthcare utilization, and expenditures through computer-assisted personal interviews and self-administered questionnaires. Respondents in each panel are interviewed for five rounds, with each round being roughly 4-6 months apart. The Household Component (HC) of MEPS, with data spanning the years 2016-2021, was used. The dataset includes yearly files such as H192, H206, H209, H216, H224, H233, and H36u21, each contributing to the comprehensive understanding of healthcare utilization, expenditures, and insurance coverage for the US civilian non-institutionalized population. MEPS has been widely used in previous opioid studies due to its comprehensive nature and national representativeness [14]. It provides detailed information on prescription medications, including opioids, along with associated diagnoses, healthcare utilization, and expenditures.

Study sample: chronic non-cancer pain patients

**Figure 1:**
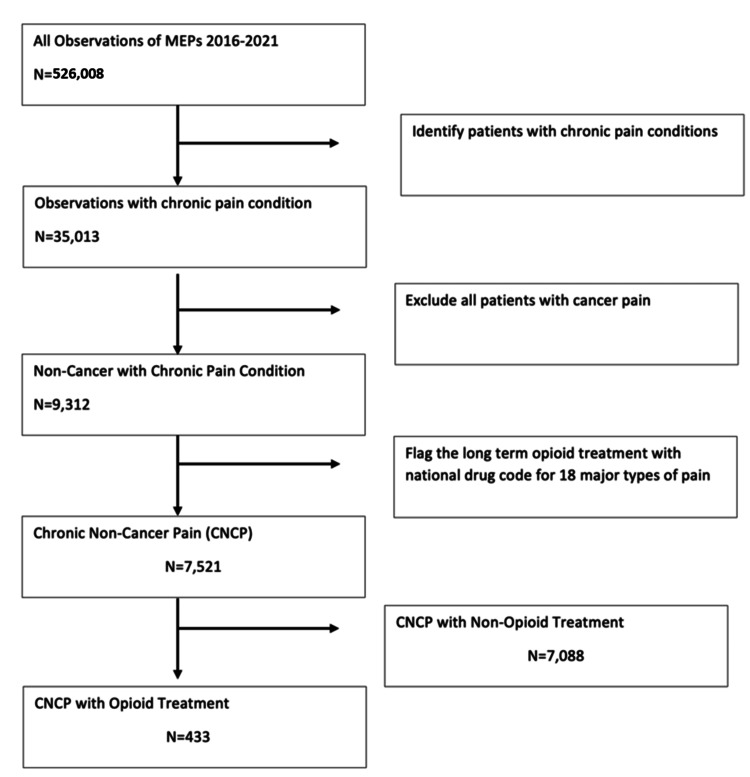
Flow diagram of study sample selection from the 2016-2021 Medical Expenditure Panel Survey (MEPS) dataset.

The participant cohort was established through the merging of various MEPS files (Figure 1). The inclusion criteria involved individuals with chronic pain conditions, identified through self-reported data and medical event records. Exclusion criteria, as per the ICD-10 diagnosis codes, ensured the removal of patients with cancer diagnoses (ICD-10 codes: 140.00-209.36, 209.70-209.79, 511.81, 789.51). These six annual datasets are amalgamated to produce a consolidated dataset.

Primary predictor: race/ethnicity

Race (White, Black, Asian/Pacific Islander, Native American, other, or multiple races) and ethnicity (Hispanic or non-Hispanic ethnicities) were obtained from self-reported MEPS data. For analytic purposes, race/ethnicity categories were constructed using non-Hispanic White individuals as the reference group, with comparisons made to non-Hispanic Black individuals and Hispanic individuals of any race.

Covariates

All independent variables (covariates) were derived from self-reported household responses in the MEPS. Covariates included age category, sex, geographic region, race/ethnicity, educational attainment, marital status, income class, and insurance coverage, as recorded in the MEPS-HC dataset. Race/ethnicity was included as a key analytic variable. Marital status, income, education, and insurance coverage were included as demographic and socioeconomic covariates. Calendar year in the pooled dataset was additionally coded as a covariate.

Descriptive analysis

Descriptive statistics and frequency tables were generated using the merged dataset, including relevant variables such as age, gender, race, education, insurance, and income. ICD-10 diagnosis codes were used to identify and categorize CNCP conditions. Chi-square tests assessed associations between opioid use and categorical variables.

Logistic regression modeling

Logistic regression models were employed for inferential analysis, adjusting for strata, cluster, and weighting. Logistic regression modeling was utilized to compare opioid and non-opioid samples, adjusting for covariates including age, gender, race, marital status, income level, education, and insurance. Adjusted odds ratios (ORs) with 95% confidence intervals (CIs) were estimated using survey-weighted logistic regression models to examine associations between race/ethnicity and opioid utilization. Models accounted for the complex survey design of MEPS by incorporating strata, cluster, and person-level weights. Wald chi-square statistics were used to assess statistical significance. Covariates included age, sex, race/ethnicity, insurance status, income level, education, marital status, and calendar year. Statistical significance was defined as p < 0.05.

## Results

The 2016-2021 MEPS data included 526,008 individuals. After excluding individuals with the prior condition of cancer and current diagnosis of cancer, there were 33,158 individuals. Using the ICD-10 code, 11,858 individuals in the final CNCP sample were identified, of which 7,521 individuals had pain prescription treatments, as indicated in Figure 1. From Table 1, among the 7,521 individuals with CNCP, 433 (5.51%) received opioid therapies, while 7,088 (94.49%) received non-opioid treatments, indicating substantially higher utilization of non-opioid therapies in this population. Age was significantly associated with treatment type, with individuals aged 51-65 demonstrating higher opioid utilization compared with other age groups (p = 0.0241). Gender-based differences in treatment utilization were not statistically significant (p = 0.4505). Racial and ethnic differences were observed, with non-Hispanic White individuals exhibiting higher treatment utilization compared with Hispanic, Non-Hispanic Black, and Non-Hispanic Other groups. Educational attainment was not significantly associated with treatment utilization (p = 0.6640), whereas significant differences were observed by insurance coverage (p = 0.0001) and income level (p = 0.0001). Individuals with private insurance and higher income levels demonstrated higher treatment utilization compared with those with public insurance or no insurance, underscoring the association between socioeconomic factors and patterns of pain management.

**Table 1:** Characteristics of the study sample at inclusion. Data are presented as n (%), unless otherwise specified. p-values were calculated using chi-square tests. Statistical significance was defined as p < 0.05.

Characteristic	Overall (N = 7,521)	Opioid users (n = 433)	Non-users (n = 7,088)	Chi-square (χ²)	p-value
Sample size	7,521 (100.00)	433 (5.51)	7,088 (94.49)		
Age (years)				9.43	0.0241
18-50	2,603 (39.87)	142 (1.92)	2,461 (37.95)		
51-65	2,377 (31.36)	171 (2.11)	2,206 (29.24)		
65-75	1,586 (17.68)	73 (0.89)	1,513 (16.78)		
75-85	955 (11.03)	47 (0.56)	908 (10.51)		
Gender				0.57	0.4505
Male	3,265 (45.61)	199 (2.62)	3,066 (42.98)		
Female	4,256 (54.39)	234 (2.88)	4,022 (51.51)		
Race/ethnicity				11.87	0.0196
Hispanic	1,114 (12.44)	66 (0.68)	1,048 (11.76)		
Non-Hispanic Black	1,048 (11.03)	71 (0.73)	977 (10.30)		
Non-Hispanic White	4,842 (68.64)	269 (3.67)	4,573 (64.97)		
Non-Hispanic Other	517 (7.87)	27 (0.41)	490 (7.46)		
Education				0.19	0.6640
High school or less	30 (0.31)	2 (0.01)	28 (0.29)		
College or more	7,491 (99.69)	431 (5.49)	7,060 (94.19)		
Insurance				18.62	0.0001
Private	4,495 (66.33)	209 (2.96)	4,286 (63.38)		
Public	2,730 (29.68)	205 (2.31)	2,525 (27.36)		
Uninsured	296 (3.97)	19 (0.22)	277 (3.75)		
Income				22.41	0.0001
Poor/negative	1,152 (10.83)	95 (0.92)	1,056 (9.92)		
Near poor	372 (4.13)	30 (0.37)	342 (3.76)		
Low income	1,042 (12.40)	74 (0.89)	968 (11.51)		
Middle income	1,968 (26.29)	114 (1.48)	1,854 (24.80)		
High income	2,988 (46.34)	120 (1.83)	2,868 (44.51)		

Based on Table 2, gender and education demonstrated no significant association with opioid therapy utilization and therefore were not included in the model (Table 1). Analysis across age groups revealed nuanced associations with opioid therapy utilization, with individuals aged 51-65 exhibiting higher estimated odds compared with other age brackets (OR = 1.341, 95% CI: 1.233-1.730, p < 0.05). However, statistical significance was not consistent across all age groups, suggesting a complex relationship between age and opioid prescribing practices. Racial disparities were observed, with non-Hispanic Black individuals demonstrating lower estimated odds of opioid therapy utilization (OR = 0.759, 95% CI: 1.240-1.686, p < 0.05), while associations for Hispanic and Non-Hispanic Other groups remained uncertain. Insurance type also showed variable effects: individuals with private insurance appeared to have lower odds of opioid therapy utilization (OR = 0.764, 95% CI: 0.421-1.387), whereas those with public insurance showed a trend toward higher odds, although statistical significance was not established for either group. Differences across income brackets suggested potential disparities in opioid therapy utilization, with lower odds observed among higher-income groups (middle income: OR = 0.446, 95% CI: 0.321-0.619; low income: OR = 0.648, 95% CI: 0.469-0.896). However, uncertainty persisted for lower-income categories, indicating the need for further investigation.

**Table 2:** Unadjusted logistic regression analysis of opioid versus non-opioid treatment for chronic non-cancer pain. Data are presented as odds ratios (OR) with 95% confidence intervals (CI). Survey-weighted unadjusted logistic regression was performed. Statistical significance was defined as p < 0.05.

Characteristic	Odds ratio (OR)	95% confidence interval
Sample size		
Unweighted sample (n)	7,521	
Weighted sample (N)	14.12M	
Age (years)		
18-50 (reference)	1	
51-65	1.341	1.233-1.730
65-75	0.937	0.643-1.366
75-85	0.99	0.658-1.490
Race/ethnicity		
Non-Hispanic White (reference)	1	
Hispanic	0.826	0.715-1.473
Non-Hispanic Black	0.759	1.240-1.686
Non-Hispanic Other	0.986	0.606-1.605
Insurance type		
Private (reference)	1	
Public	0.764	0.421-1.387
Uninsured	1.388	0.763-2.524
Income level		
High income (reference)	1	
Middle income	0.446	0.321-0.619
Low income	0.648	0.469-0.896
Near poor	0.841	0.579-1.221
Poor/negative income	1.087	0.628-1.879

Table 3 summarizes the results of the analysis performed between opioid and non-opioid treatments for NCNP. 

**Table 3:** Adjusted logistic regression analysis of opioid vs. non-opioid treatment for non-cancer chronic pain Data are presented as adjusted odds ratios (ORs) with 95% confidence intervals (CIs). Survey-weighted logistic regression was performed. Statistical significance was defined as p < 0.05. *Statistically significant.

Variable	Adjusted odds ratio (OR)	95% CI
Hispanic	1.1	0.90-1.40
Non-Hispanic Black	0.7	0.50-0.90*
Non-Hispanic Other	1	0.80-1.30

## Discussion

Among the 7,521 individuals with CNCP, 433 (5.51%) received opioid therapy, while 7,088 (94.49%) received non-opioid treatments. The decrease in opioid prescribing observed in this study is consistent with national data showing a marked overall decline among individuals with CNCP, with approximately one-third experiencing a reduction in opioid prescribing compared with the prior year in the United States [15]. Non-opioid therapies accounted for the majority of treatment utilization (94.49%). Age was significantly associated with treatment type, with higher opioid utilization observed among individuals aged 51-65 years. Gender-based differences in utilization were minimal, and educational attainment was not significantly associated with treatment utilization. In contrast, insurance coverage and income level were significantly associated with treatment utilization, with individuals holding private insurance and those in higher income brackets demonstrating greater utilization compared with those with public insurance or no insurance.

This study also underscored persistent racial disparities in opioid therapy utilization. Despite overall decreases in opioid prescribing, we found no evidence that racial or ethnic differences in opioid utilization have narrowed over time. Non-Hispanic White individuals exhibited higher opioid therapy utilization compared with Hispanic, Non-Hispanic Black, and Non-Hispanic Other groups. These findings align with prior research showing higher opioid use among Non-Hispanic White individuals (11.9%) compared with Non-Hispanic Black (9.3%) and Hispanic (9.6%) populations [16]. This contrast highlights potential differences in access to care or prescribing practices across racial groups. Additionally, lower estimated odds of opioid therapy utilization were observed among Non-Hispanic Black individuals, suggesting disparities in treatment patterns or access. Associations for Hispanic and Non-Hispanic Other groups remained uncertain, warranting further investigation to better understand the complex relationship between race and opioid therapy utilization. Importantly, these observed differences should not be interpreted as indicators of treatment preference, clinical appropriateness, or inequitable care in the absence of data on pain severity, clinical indication, or provider decision-making.

Study limitations

This study has several limitations. First, the cross-sectional design of the MEPS limits causal inference regarding differences in opioid utilization. Second, chronic pain conditions and treatment utilization were based on self-reported data, which may be subject to recall bias. Third, MEPS does not capture pain severity, duration, or the clinical appropriateness of opioid prescribing, limiting assessment of treatment adequacy. Finally, misclassification related to diagnostic coding may have occurred. Despite these limitations, the use of a nationally representative dataset enhances the generalizability of the findings.

## Conclusions

The study findings indicate meaningful differences in therapy utilization across age, race, insurance coverage, and income groups, highlighting the influence of sociodemographic factors on pain management patterns. These results underscore the importance of ensuring equitable access to a range of pain management options and avoiding one-size-fits-all approaches to CNCP care. Importantly, observed differences in utilization likely reflect complex, multifactorial processes rather than uniform treatment preferences. Future research should incorporate additional clinical and contextual factors and apply more robust analytic approaches, such as multivariable regression and longitudinal designs, to better elucidate the relationship between sociodemographic characteristics and therapy utilization. Addressing these disparities remains essential for informing patient-centered and equity-oriented pain management strategies.
